# Association between interleukin-10 gene polymorphisms (rs1800871, rs1800872, and rs1800896) and severity of infection in different SARS-CoV-2 variants

**DOI:** 10.1186/s40246-023-00468-6

**Published:** 2023-03-07

**Authors:** Sattar Jabbar Abbood Abbood, Enayat Anvari, Abolfazl Fateh

**Affiliations:** 1 Department of Biology, Science and Research Branch, Islamic Azad University, Tehran, Iran; 2 Clinical Research Development Unit, Shahid Mostafa Khomeini Hospital, Ilam University of Medical Science, Ilam, Iran; 3 Department of Mycobacteriology and Pulmonary Research, Pasteur Institute of Iran, Tehran, Iran; 4 Microbiology Research Center (MRC), Pasteur Institute of Iran, Tehran, Iran

**Keywords:** COVID-19, SARS-CoV-2 variants, Interleukin-10 gene polymorphisms

## Abstract

**Background:**

Polymorphisms in the interleukin-10 (*IL10*) gene have been linked to the severity of the patients infected with the viral infections. This study aimed to assess if the *IL10* gene polymorphisms rs1800871, rs1800872, and rs1800896 were linked to coronavirus disease 19 (COVID-19) mortality in different severe acute respiratory syndrome coronavirus 2 (SARS-CoV-2) variants in the Iranian population.

**Methods:**

For genotyping *IL10* rs1800871, rs1800872, and rs1800896, this study used the polymerase chain reaction-restriction fragment length polymorphism method in 1,734 recovered and 1,450 deceased patients.

**Results:**

The obtained finding indicated *IL10* rs1800871 CC genotype in the Alpha variant and CT genotype in the Delta variant had a relationship with COVID-19 mortality; however, there was no association between rs1800871 polymorphism and the Omicron BA.5 variant. The COVID-19 mortality rate was associated with *IL10* rs1800872 TT genotype in the Alpha and Omicron BA.5 variants and GT in the Alpha and Delta variants. The COVID-19 mortality rate was associated with *IL10* rs1800896 GG and AG genotypes in the Delta and Omicron BA.5; nevertheless, there was no association between rs1800896 polymorphism with the Alpha variant. According to the obtained data, the GTA haplotype was the most common of haplotype in different SARS-CoV-2 variants. The TCG haplotype was related to COVID-19 mortality in the Alpha, Delta and Omicron BA.5 variants.

**Conclusion:**

The *IL10* polymorphisms had an impact on COVID-19 infection, and these polymorphisms had different effects in various SARS-CoV-2 variants. To verify the obtained results, further studies should be conducted on various ethnic groups.

## Introduction

The coronavirus disease 2019 (COVID-19) pandemic has been a persistent threat to public health for over a few years. Severe acute respiratory syndrome coronavirus 2 (SARS-CoV-2) variations that are more fatal and contagious are a serious worry, especially as causative therapy is still lacking and vaccination coverage rates are lower than expected. Additionally, vaccination-induced immunity is probably only going to last one or two seasons. Identifying populations at high risk of developing severe COVID-19 and putting protective measures in place for those populations is one way to save lives. The COVID-19 mortality toll was the highest in the elderly, obese, male, immunocompromised, tobacco users, chronic disease patients, socioeconomically disadvantaged, black people, and cancer [1, 2]. Additionally, interindividual genetic differences could be a factor in COVID-19 cases that are more severe [3–9].

Recent research has revealed that the amount of inflammatory cytokines is elevated in COVID-19. According to a literature review,, interleukins-2** (**IL2), IL6, IL7, IL10, granulocyte colony-stimulating factor (G-CSF), interferon gamma, inducible protein-10, tumor necrosis factor alpha, monocyte chemoattractant protein-1, macrophage inflammatory protein-1 all play important roles in COVID-19 development [10, 11].

The IL-10, known as a pleiotropic cytokine, has strong immunosuppressive and anti-inflammatory effects. Initially, it was thought that IL-10 was produced by T helper 2 cells; it is currently understood that IL-10 is produced by a variety of immune cells with lymphoid and myeloid origins that function in both adaptive and innate immunity [12]. Several studies show that high IL-10 expression levels predict poor outcomes in patients with COVID-19 and appear to be a distinguishing feature of hyperinflammation during severe SARS-CoV-2 infection. The IL-10 is canonically categorized as an anti-inflammatory cytokine and rises dramatically early in the course of the disease [13, 14].

Three polymorphisms, *IL10* rs1800871 (− 819 T/C), rs1800872 (− 592 C/A), and rs1800896 (-1082 G/A), in the promoter region of *IL10* gene have been studied more to date. Their haplotypes in different populations are related to the low or high expression of *IL10* gene. Polymorphisms in the promoter region contribute genetically to interindividual variations in IL10 production. The *IL10* rs1800896 (-1082 G/A) polymorphism has been found to be associated with greater IL10 serum levels and an increased risk of developing severe pneumonia [15]. Additionally, the *IL10* rs1800872 (592 C > A) polymorphism of the gene causes a considerable reduction in the negative promoter function, changing *IL10* transcription and mRNA production [16].

Concerning the efficacy of IL10 in regulating T-cell activity and its effects on viral infections, in this study examined three single-nucleotide polymorphisms** (**SNPs) in the *IL10* promoter (rs1800871, rs1800872, and rs1800896) to determine how host genetic variables affect COVID-19 severity according SARS-CoV-2 variants.

## Materials and methods

### Patients definition

The current study comprised 3,184 patients with a diagnosis of COVID-19 who were referred to a teaching hospital of Ilam University of Medical Sciences, Ilam, Iran, within November 2020 to February 2022, including 1,734 recovered and 1450 deceased patients. A COVID-19 infection was deemed for all patients as a result of a positive SARS-CoV-2 laboratory test with real-time reverse transcription polymerase chain reaction (rtReal time-PCR) from the nasopharyngeal swabs. Peripheral blood samples from each patient were taken to isolate deoxyribonucleic acid (DNA) and conduct additional genetic studies.

The samples were collected in the three peaks (Alpha, Delta, and Omicron BA.5) from 14,472 positive patients based on the inclusion criteria, namely (1) patients who were willing to participate in the study and had signed a written consent form, (2) all patients who were Iranian with one ethnicity, and (3) patients who did not have any underlying comorbidities diseases, such as kidney, heart, and pulmonary diseases, hypertension, diabetes, obesity, cancer, viral infections (e.g., human immunodeficiency virus and hepatitis B and C viruses), and pregnancy.

All clinical data of patients such as real-time PCR cycle threshold (Ct) values, 25-hydroxyvitamin D, C-reactive protein (CRP), erythrocyte sedimentation rate (ESR), complete blood count (CBC), lipid profile (cholesterol, high density lipoprotein, and low density lipoprotein), liver enzymes (aspartate aminotransferase, alkaline phosphatase, and alanine aminotransferase), creatinine and uric acid were extracted from patient files, and these tests were performed when the patient entered the hospital.

According to the World Health Organization guidelines, adult COVID-19 patients were divided into three clinical course categories, mild, moderate, and severe. In this study, subjects with mild/moderate and severe/critical symptoms were considered recovered and deceased patients, respectively.

Patients with mild symptoms include those who have a fever, fatigue, cough, headache, myalgia, and fatigue but do not have dyspnea or pneumonia; patients with moderate symptoms include those who have blood oxygen saturation levels above 93% on room air and evidence of pneumonia based on imaging showing up to 50% lung involvement; patients with severe symptoms include those who have blood oxygen saturation levels below 93% on room air and need supportive oxygen therapy.

### *IL10* rs1800871, rs1800872, and rs1800896 genotyping

After genomic DNA isolation of all patients using QIAamp DNA Blood Mini Kit (Qiagen, Hilden, Germany), *IL10* rs1800871, rs1800872, and rs1800896 genotyping was carried out by polymerase chain reaction-restriction fragment length polymorphism (PCR–RFLP) technique.

The PCR was conducted according to the following conditions: initial denaturation at 95ºC for 5 min, 37 cycles of 95ºC for 30 s, 57 ºC (rs1800896 and rs1800871) and 61ºC (rs1800872) for 40 s, 72ºC for 45 s, and final extension at 72ºC for 10 min. The specific primers for each location were as follows: for *IL10* rs1800871 was f-5'-CTCGCCGCAACCCAACTGGC-3' and r-5'-TCTTACCTATCCCTACTTCC-3' and for rs1800872 was f-5'-GGTGAGCACTACCTGACTAGC-3' and r-5'-CCTAGGTCACAGTGACGTGG-3', and for rs1800896 was f-5'-CTCGCCGCAACCCAACTGGC-3' and r-5'-TCTTACCTATCCCTACTTCC-3'. In a 25 ml reaction mixture, RFLP digestion was carried out using *RseI* (Thermo, USA), *RsaI* (New England Biolabs, USA), and *MnII* (New England Biolabs, USA) for the rs1800871, rs1800872, and rs1800896, respectively. The reaction mixture was incubated at 37 °C for 16–18 h before being separated on a 2% agarose gel electrophoresis.

The undigested PCR products with 209-bp for *IL10* rs1800871 represented the T allele. The existence of the C allele was established by visualizing two 125- and 84-bp-sized PCR product fragments that had been digested. The C allele was represented by the 412-bp PCR products of rs1800872. Observing two 176- and 236-bp long fragments of the digested PCR result verified the existence of the A allele. Finally, the T allele was represented by the 134-bp undigested PCR product of rs1800896. The G allele’s presence was verified by visualizing two digested PCR product fragments with 101- and 33-bp [17]. Several samples were randomly chosen and sequenced using the Sanger sequencing method to corroborate the PCR–RFLP results.

### Statistical analyses

Statistical analysis was conducted in SPSS version 22.0 software (SPSS, Inc, Chicago, IL, USA). Using appropriate statistical analyses for continuous and discrete data (the Mann–Whitney U test and Chi-square tests), differences in demographic and clinical data between COVID-19 recovered and deceased groups were investigated. The Hardy–Weinberg equilibrium (HWE) was investigated using genetic data to evaluate the effectiveness of the genotyping tests. The Chi-squared test was used to compare genotype and allele count distributions among COVID-19 subgroups for each variant. In order to account for confounding variables, including SARS-CoV-2 variants, the effect of each genetic trait on the severity of COVID-19 was assessed by odds ratio (OR) with a 95% confidence interval (CI) using a logistic regression model. The correlation study was performed using the SNPStats program, which also allowed for the determination of the minor allele frequency (MAF), HWE and dominant, over-dominant, co-dominant, and recessive models. The Akaike Information Criterion (AIC) and the Bayesian Information Criterion (BIC) were used to determine which model suited the data the best. The model with the lowest AIC score was the one that performed the best (http://bioinfo.iconcologia.net/SNPStats). Statistics were considered significant at *P*-values less than 0.05.

## Results

### Demographics and baseline clinical characteristics of COVID-19 patients

According to Table 1, this study included three variants, the Alpha, Delta, and Omicron with 1,022, 1,026, and 1,132 patients, respectively. The Alpha (53.0 ± 12.7) and the Omicron BA.5 (53.7 ± 12.9) variants were both younger than the Delta variant (58.0 ± 11.8). In the Alpha variant, there were 479 (46.9%) male and 543 (53.1%) female. In the Delta variant, there were 546 (53.2%) male and 480 (46.8%) female patients. In the Omicron BA.5 variant, there were also 546 (53.2%) male and 480 (46.8%) female patients.

**Table 1:** Comparison of laboratory parameters between SARS-CoV-2 variants

	SARS-CoV-2 variants			
Variables	Alpha (*n* = 1,022)	Delta (*n* = 1,026)	Omicron BA.5 (*n* = 1,136)	*P*-value
Deceased/ Improved patients	479/543 (46.9/53.1%)	674/352 (65.7/34.3%)	297/839 (26.1/73.9%)	< 0.001*
Mean age ± SD	53.0 ± 12.7	58.0 ± 11.8	53.7 ± 12.9	0.128
Gender (male/female)	525/497(51.4/48.6%)	546/480 (53.2/46.8%)	598/538 (52.6/47.4%)	0.692
ALT, IU/L (mean ± SD) (Reference range: 5–40)	38.5 ± 24.8	40.8 ± 24.7	35.8 ± 24.2	0.001
AST, IU/L (mean ± SD) (Reference range: 5–40)	34.9 ± 15.5	34.5 ± 14.0	31.9 ± 14.4	< 0.001*
ALP, IU/L (mean ± SD) (Reference range: up to 306)	190.2 ± 84.7	188.6 ± 74.0	177.2 ± 83.5	< 0.001*
Cholesterol, mg/dL (mean ± SD) (Reference range: 50–200)	116.1 ± 34.1	120.5 ± 40.5	123.1 ± 39.4	< 0.001*
TG, mg/dL (mean ± SD) (Reference range: 60–165)	124.1 ± 54.9	121.6 ± 48.8	126.9 ± 55.9	0.245
LDL, mg/dL (mean ± SD) (Reference range: up to 150)	82.8 ± 45.1	85.3 ± 45.3	104.7 ± 48.3	< 0.001*
HDL, mg/dL (mean ± SD) (Reference range: > 40)	32.5 ± 11.3	32.1 ± 11.5	33.6 ± 11.7	0.039*
WBC, 10^9^/L (mean ± SD) (Reference range: 4000–10,000)	7627.3 ± 2843.2	7599.2 ± 2715.7	7704.9 ± 2807.7	0.297
CRP, mg/L (mean ± SD) (Reference range: < 10 mg/L Negative)	61.6 ± 21.5	63.9 ± 22.0	60.2 ± 21.7	0.122
ESR, mm/1st h (mean ± SD) (Reference range: 0–15)	50.1 ± 16.0	52.3 ± 16.0	49.1 ± 16.1	0.025
FBS, mg/dL (mean ± SD) (Reference range: 70–100)	107.1 ± 41.6	109.8 ± 43.2	106.5 ± 40.7	0.716
Platelets × 1000/cumm (mean ± SD) (Reference range: 140,000–400,000)	184 ± 71	185 ± 74	184 ± 69	0.994
Uric acid, mg/dL (mean ± SD) (Reference range: 3.6–6.8)	4.8 ± 1.8	4.4 ± 1.7	5.2 ± 1.8	< 0.001*
Creatinine, mg/dL (mean ± SD) (Reference range: 0.6–1.4)	0.9 ± 0.3	1.0 ± 0.3	0.8 ± 0.3	< 0.001*
qPCR Ct value	20.1 ± 6.4	17.4 ± 6.1	21.9 ± 6.0	< 0.001*
25-hydroxy vitamin D, ng/mL (mean ± SD) (Sufficiency: 21–150)	24.2 ± 12.8	21.8 ± 10.3	33.0 ± 13.4	0.029*
*IL10* rs1800871				< 0.001*
TT	271 (26.5%)	498 (48.5%)	391 (34.4%)	
CT	550 (53.8%)	377 (36.7%)	619 (54.5%)	
CC	201 (19.7%)	151 (14.8%)	126 (11.1%)	
*IL10* rs1800872				< 0.001*
GG	474 (46.4%)	532 (51.9%)	269 (23.7%)	
GT	396 (38.7%)	386 (37.6%)	711 (62.6%)	
TT	152 (14.9%)	108 (10.5%)	156 (13.7%)	
*IL10* rs1800896				< 0.001*
AA	358 (35.0%)	510 (49.7%)	575 (50.6%)	
AG	550 (53.8%)	338 (32.9%)	511 (45.0%)	
GG	114 (11.2%)	178 (17.4%)	50 (4.4%)	

*ALT* Alanine aminotransferase, *AST* Aspartate aminotransferase, *ALP* Alkaline phosphatase, *TG* Triglyceride, *LDL* Low density lipoprotein, *HDL* High density lipoprotein, *WBC* White blood cells, *CRP* C-reactive protein, *ESR* Erythrocyte sedimentation rate, *FBS* Fasting blood glucose, *SD* Standard deviation, *SARS-CoV-2* Severe Acute Respiratory Syndrome Coronavirus 2, *IL10* interleukins 10. *Statistically significant (< 0.05)

The 25-hydroxy vitamin D rate was significantly different between the Alpha, Delta, and Omicron BA.5 variants (*P* = 0.029) and was (24.2 ± 12.8, 21.8 ± 10.3, and 33.0 ± 13.4), respectively. Compared to the Alpha (20.1 ± 6.4) and Omicron BA.5 (21.9 ± 6.0) variants, the mean qPCR Ct values in the Delta variation (17.4 ± 6.1) were greater (*P* < 0.001).

### COVID-19 mortality adjusted by SARS-CoV-2 variants and *IL10* polymorphisms rs1800871, rs1800872, and rs1800896

The *IL10* rs1800871 CC genotype, compared to other genotypes, was significantly related to COVID-19 mortality. In *IL10* rs1800872 and rs1800896 polymorphisms, the patients with TT and GG genotypes had a higher COVID-19 death rate.

Table 2 shows the findings of the inheritance model analysis for *IL10* rs1800871, rs1800872, and rs1800896 polymorphisms in patient samples. The Codominant model for all three SNPs with the lowest AIC and BIC in studied patients was the best fitting ones. The *IL10* rs1800871 CC genotype was correlated with a higher risk of COVID-19 mortality (*P* < 0.0001, OR 3.59, 95% CI 2.82–4.56). In *IL10* rs1800872 TT genotype (*P* < 0.0001, OR 3.39, 95% CI 2.65–4.35) and GG in rs1800896 (*P* < 0.0001, OR 2.65, 95% CI 2.04–3.45) were correlated to a higher risk of COVID-19 mortality.

**Table 2:** *IL10* gene polymorphisms association with COVID-19 mortality adjusted by SARS- CoV-2 variants

*IL10* rs1800871		Groups					
Model	Genotype	Recovered patients	Deceased patients	OR (95% CI)	*P*-value	AIC	BIC
Allele	T	2281 (66.0%)	1585 (55.0%)	–	–	–	–
	C	1187 (34.0%)	1315 (45.0%)	–	–	–	–
Codominant	T/T	716 (41.2%)	444 (30.6%)	1.00	< 0.0001*	3927.2	3957.6
	C/T	849 (49.0%)	697 (48.1%)	1.78 (1.50–2.12)			
	C/C	169 (9.8%)	309 (21.3%)	3.59 (2.82–4.56)			
Dominant	T/T	716 (41.2%)	444 (30.6%)	1.00	< 0.0001*	3963.5	3987.7
	C/T- C/C	1018 (58.8%)	1006 (69.4%)	2.10 (1.79–2.48)			
Recessive	T/T-C/T	1565 (90.2%)	1141 (78.7%)	1.00	< 0.0001*	3970.1	3994.3
	C/C	169 (9.8%)	309 (21.3%)	2.53 (2.04–3.13)			
Overdominant	T/T-C/C	885 (51.0%)	753 (51.9%)	1.00	0.023	4040.6	4064.8
	C/T	849 (49.0%)	697 (48.1%)	1.19 (1.02–1.38)			
Minor allele frequency (C)		0.34	0.45	–	–	–	–
*IL10* rs1800872							
Allele	G	2290 (66.0%)	1753 (60.0%)	–	–	–	–
	T	1178 (34.0%)	1147 (40.0%)	–	–	–	–
Codominant	G/G	703 (40.5%)	572 (39.5%)	1.00	< 0.0001*	3948.0	3978.3
	G/T	884 (51.0%)	609 (42.0%)	1.25 (1. 06–1.48)			
	T/T	147 (8.5%)	269 (18.6%)	3.39 (2.65–4.35)			
Dominant	G/G	703 (40.5%)	572 (39.5%)	1.00	< 0.0001*	4014.9	4039.1
	G/T-T/T	1031 (59.5%)	878 (60.5%)	1.56 (1.33–1.83)			
Recessive	G/G-G/T	1587 (91.5%)	1181 (81.5%)	1.00	< 0.0001*	3953.0	3977.2
	T/T	147 (8.5%)	269 (18.6%)	2.99 (2.38–3.75)			
Overdominant	G/G-T/T	850 (49.0%)	841 (58.0%)	1.00	0.21	4044.2	4068.45
	G/T	884 (51.0%)	609 (42.0%)	0.91 (0.78–1.06)			
Minor allele frequency (T)		0.34	0.40	–	–	–	–
*IL10* rs1800896							
Allele	A	2530 (73.0%)	1755 (61.0%)	–	–	–	–
	G	938 (27.0%)	1145 (39.0%)	–	–	–	–
Codominant	A/A	912 (52.6%)	531 (36.6%)	1.00	< 0.0001*	3954.1	3984.4
	A/G	706 (40.7%)	693 (47.8%)	1.95 (1. 66–2.29)			
	G/G	116 (6.7%)	226 (15.6%)	2.65 (2.04–3.45)			
Dominant	A/A A/G-G/G	912 (52.6%)	531 (36.6%)	1.00	< 0.0001*	3957.5	3981.8
		822 (47.4%)	919 (63.4%)	2.07 (1.78–2.42)			
Recessive	A/A-A/G	1618 (93.3%)	1224 (84.4%)	1.00	< 0.0001*	4018.6	4042.8
	G/G	116 (6.7%)	226 (15.6%)	1.91 (1.49–2.44)			
Overdominant	A/A-G/G	1028 (59.3%)	757 (52.2%)	1.00	< 0.0001*	4007.9	4032.2
	A/G	706 (40.7%)	693 (47.8%)	1.61 (1.38–1.88)			
Minor allele frequency (G)		0.27	0.39	–	–	–	–

*COVID-19* Coronavirus disease, *OR* Odds ratios, *CI* Confidence intervals, *IL10* Interleukins 10, *AIC* Akaike information criterion, *BIC* Bayesian information criterion, *OR* Odds ratios, *CI* Confidence intervals; *Statistically significant (< 0.05)

The *IL10* rs1800871 (*P* = 0.33), rs1800872 (*P* = 0.54), and rs1800896 (*P* = 0.94) polymorphisms in recovered and deceased patients were compatible with the HWE. The MAF for *IL10* rs1800871 (C), rs1800872 (T) and rs1800896 (G) polymorphisms in recovered patients was lower than those in recovered ones.

### *IL10* polymorphisms rs1800871, rs1800872, and rs1800896 frequencies in SARS-CoV-2 variants

The results of this study showed that the mortality rate was significantly higher in the Delta variant than in the other two variants (*P* < 0.001).

Table 1 lists the frequency of *IL10* rs1800871, rs1800872, and rs1800896 genotypes in different SARS-CoV-2 variants. Briefly, in *IL10* rs1800871 polymorphism, the frequency of TT, CT, and CC in the Alpha variant was 271 (26.5%), 550 (53.8%), and 201 (19.7%), respectively. In the Delta variant, the frequencies were 498 (48.5%), 377 (36.7%), and 151 (14.8%), respectively. In the Omicron variant, the frequencies were 391 (34.4%), 619 (54.5%), and 126 (11.1%), respectively.

In *IL10* rs1800872 polymorphism, the frequency of GG, GT, and TT in the Alpha variant was 474 (46.4%), 396 (38.7%), and 152 (14.9%), respectively. In the Delta variant was 532 (51.9%), 386 (37.6%), and 108 (10.5%) and in the Omicron BA.5 was 269 (23.7%), 711 (62.6%), and 156 (13.7%), respectively.

In *IL10* rs1800896 polymorphism, the frequency of AA, AG, and GG in the Alpha variant was 358 (35.0%), 550 (53.8%), and 114 (11.2%), respectively. In the Delta variant was 510 (49.7%), 338 (32.9%), and 178 (17.4%) and in the Omicron BA.5 was 575 (50.6%), 511 (45.0%), and 50 (4.4%), respectively (Table 1).

After adjusting the association of *IL10* rs1800871 polymorphism with SARS-CoV-2 variants, the CC genotype (OR 3.92, 95% CI 2.64–5.82) in the Alpha variant and CT genotype (OR 1.32, 95% CI 1.01–1.73) in the Delta variant had a relationship with COVID-19 mortality; however, there was no association between rs1800871 polymorphism with the Omicron BA.5 variant (Table 3).

**Table 3:** *IL10* rs1800871, rs1800872, and rs1800896 genotypes association with SARS-CoV-2 variants

Variants	rs1800871 Genotypes	Recovered patients	Deceased patients	OR (95% CI)
Alpha	T/T	160	111	1.00
	C/T	329	221	0.97 (0.72–1.30)
	C/C	54	147	3.92 (2.64–5.82)
Delta	T/T	165	333	1.00
	C/T	101	276	1.35 (1.01–1.82)
	C/C	86	65	–
Omicron BA.5	T/T	419	1	1.00
	C/T	29	199	–
	C/C	38	97	–
Variants	rs1800872 Genotypes	Recovered patients	Deceased patients	OR (95% CI)
Alpha	G/G	281	193	1.00
	G/T	199	197	1.44 (1.10–1.89)
	T/T	63	89	2.06 (1.42–2.98)
Delta	G/G	224	308	1.00
	G/T	76	310	2.97 (2.19–4.02)
	T/T	52	56	0.78 (0.52–1.19)
Omicron BA.5	G/G	198	71	1.00
	G/T	602	102	–
	T/T	32	124	10.81 (6.73–17.36)
Variants	rs1800896 Genotypes	Recovered patients	Deceased patients	OR (95% CI)
Alpha	A/A	182	176	1.00
	A/G	308	242	0.81 (0.62–1.06)
	G/G	53	61	1.19 (0.78–1.82)
Delta	A/A	211	299	1.00
	A/G	107	231	1.52 (1.14–2.03)
	G/G	34	144	2.99 (1.98–4.52)
Omicron BA.5	A/A	519	56	1.00
	A/G	291	220	7.01 (5.05–9.71)
	G/G	29	21	6.71 (3.59–12.55)

*SARS-CoV-2* Severe Acute Respiratory Syndrome Coronavirus 2, *IL10* Interleukins 10, *OR* Odds ratios, *CI* Confidence intervals

The COVID-19 mortality rate was associated with *IL10* rs1800872 TT genotype in the Alpha (OR 2.06, 95% CI 1.42–2.98) and Omicron BA.4 (OR 10.81, 95% CI 6.73–17.36) variants and GT in the Alpha (OR 1.44, 95% CI 1.10–1.89*)* and Delta (OR 2.97, 95% CI 2.19–4.02*)* variants (Table 3).

The COVID-19 mortality rate was associated with *IL10* rs1800896 GG genotype in the Delta (OR 1.52, 95% CI 1.14–2.03) and Omicron BA.4 (OR 6.71, 95% CI 3.59–12.55) variants and AG in the Delta (OR 2.99, 95% CI 1.98–4.52*)* and Omicron BA.4 (OR 7.01, 95% CI 5.05–9.71*)* variants; however, there was no association between rs1800896 polymorphism with the Alpha variant (Table 3).

According to the obtained data of the current study, the GTA haplotype was the most common of haplotype in different SARS-CoV-2 variants. The TCG haplotype was related to COVID-19 mortality in the Alpha (OR 1.34, 95% CI 1.09–1.65*)*, Delta (OR 1.33, 95% CI 1.06–1.67*)* and Omicron BA.5 (OR 35.92, 95% CI 21.84–59.07*)* variants. The likelihood of death in patients with the Omicron BA.5 variant was 35-fold, compared to other variants. The GCA haplotype for the Alpha variant (OR 8.02, 95%CI 4.91–13.08) was statistically significant. The TCA haplotype for the Alpha (OR 58.26, 95%CI 7.48–79.96) and Omicron BA.5 (OR 19.44, 95%CI 11.15–33.87) variants was observed as statistically significant. The TTA and GCG haplotypes were associated with the mortality rate in the Omicron BA.5 (OR 7.08, 95%CI 3.73–13.44) and Delta (OR 3.55, 95%CI 1.28–9.88) variants, respectively (Table 4).

**Table 4:** SARS-CoV-2 variants and *IL10* rs1800871, rs1800872, and rs1800896 haplotypes

		Alpha	Delta	Omicron
Haplotypes	Frequency	OR (95% CI)	OR (95% CI)	OR (95% CI)
GTA	0.5486	1.00	1.00	1.00
TCG	0.2817	1.34 (1.09–1.65)	1.33 (1.06–1.67)	35.92 (21.84–59.07)
GCA	0.0443	8.02 (4.91–13.08)	–	–
TCA	0.0415	58.26 (7.48–79.96)	–	19.44 (11.15–33.87)
TTA	0.0384	–	–	7.08 (3.73–13.44)
GCG	0.0253	–	3.55 (1.28–9.88)	–
GTG	0.0166	–	–	–

*SARS-CoV-2* Severe Acute Respiratory Syndrome Coronavirus 2, *IL10* Interleukins 10, *OR* Odds ratios, *CI* Confidence intervals

## Discussion

The current study used different SARS-CoV-2 variants to examine the genetic susceptibility of the host to COVID-19 mortality. To ascertain if *IL10* rs1800871, rs1800872, and rs1800896 polymorphisms are connected to the vulnerability to COVID-19 mortality according to different SARS-CoV-2 variants, the alleles and their genotypes were investigated.

In patients with COVID-19, the allele C (0.36) for the *IL10* rs1800871 as MAF was directly correlated with death. This amount was equal to Asian (0.317), other Asian (0.379), and East Asian (0.293) and was different from other regions in European (0.738), African (0.594), and South Asian (0.585) (https://www.ncbi.nlm.nih.gov/snp/rs1800871). In this study, the MAF for *IL10* rs1800871 in recovered patients (0.34) was lower than in the deceased ones (0.45).

In patients with COVID-19, the allele T (0.37) for the *IL10* rs1800872 as MAF was directly correlated with death. This amount was equal to Iran, South Asian (0.380), Latin American (0.361), African American (0.407), and European (0.293) and was different from other regions in Asian (0.726), East Asian (0.760), other Asian (0.610) and African (0.408) (https://www.ncbi.nlm.nih.gov/snp/rs1800872). In this study, the MAF for *IL10* rs1800872 in recovered patients (0.34) was lower than in the deceased ones (0.40).

The MAF for *IL10* rs1800896 (C) was 0.39 that was almost similar to Iran, South Asian (0.290), African (0.331), African American (0.334), Latin American (0.356), and European (0.473); however, it was different from Asian (0.063), East Asian (0.060), and other Asian (0.074) (https://www.ncbi.nlm.nih.gov/snp/rs1800896). In this study, the MAF for *IL10* rs1800896 in recovered patients (0.27) was lower than in the deceased ones (0.39).

There have been reports of a link between SNPs in the *IL10* gene and respiratory viral infectious diseases; this cytokine is thought to be a critical molecule in COVID-19 development. Due to this issue, the present study offered to examine, for the first time, if the COVID-19 death rate is connected with the polymorphisms rs1800871, rs1800872, and rs1800896 in a cohort of Iranian patients infected with different SARS-CoV-2 variants. These polymorphisms are a member of a collection of haplotypes linked to various amounts of IL10 production [18, 19]. In a study has been shown that Omicron variant showed lower IL-10 concentrations compared to other variants, a notion that can potentially be explained by clinical features of this specific variant [20].

In this study, the COVID-19 mortality rate was associated with the *IL10* rs1800896 GG and AG genotypes in the Delta and Omicron BA.4 variants; nevertheless, there was no association between rs1800896 polymorphism with the Alpha variant. Rizvi et al., indicated that AG genotypes was correlated with COVID-19 severity [21]. The G to A polymorphism at rs1800896 controls how the *IL10* gene is expressed. It has been reported that individuals with the GG genotype have higher levels of *IL10* transcription and circulating levels of IL10 than individuals with the AA genotype [22]. According to a study involving 23 countries, there is a substantial positive connection between the frequency of the rs1800896 AG genotype and the prevalence of COVID-19. The *IL10* gene polymorphisms in different populations at the rs1800896 locus revealed that populations in Japan, China, Tunisia, and Mexico frequently have the AA genotype; however, populations in Iran, India, the Netherlands, Finland, Germany, Spain, Czechia, Norway, Poland, the UK, and Brazil frequently have the AG genotype. Only among the Italian population the rs1800896 GG genotype had the highest frequency [23]. Moreover, the *IL10* rs1800896 AG genotype was substantially related to death in infections with influenza A/H1N1pdm09 [24]. Due to the increased expression of the *IL10* gene, it seems that G allele as MAF compared to A allele plays an important role in the susceptibility to severe COVID-19 infection. However, which allele can play a role in the infection of COVID-19 can depend on various factors such as race.

The rs1800896 AG and GG genotypes are linked to an increased risk of hepatitis B virus (HBV) and can make individuals more vulnerable to it. On the other side, chronic HBV patients have been shown to have elevated levels of IL10, suggesting that those with the rs1800896 G allele are at risk for contracting HBV [25]. Additionally, the rs1800896 GG genotype was correlated with the increased risk of systemic lupus erythematosus [17].

The findings of the present study revealed that the COVID-19 mortality rate was associated with the *IL10* rs1800872 TT genotype in Alpha and Omicron variants and GT in Alpha and Delta variants. The GG genotype was observed to play a substantial protective function in preventing COVID-19 severity among patients who carried the rs1800872 polymorphism. The frequency of the GG genotype was higher in mild than in severe COVID-19 individuals, according to a study on the Mexican population. However, the results were not determined to be statistically significant [18, 20]. The *IL10* rs1800872 GT genotypes were linked to a higher risk of contracting the influenza A/H3N2 virus. This might be a result of IL-10’s anti-inflammatory properties, which stop the natural killer and T cell activities from having an impact on the intense inflammatory response following the initial infection [26].

The *IL10* rs1800872 polymorphism is linked to an increase in the severity of autoimmune and infectious diseases and regulates the transcription and production of IL10. The rs1800872 TT genotype was related to rheumatoid arthritis susceptibility in Iranian patients [27]. Studies from other cultures, such as Hong Kong and China, ruled out the link between this polymorphism and susceptibility and severity of other viral illnesses, such as influenza A/H1N1pdm09 and SARS [28]. The inconsistent findings in these kinds of studies can be addressed from the perspectives of immunogenetics and population genetics by taking into account various human immune responses to viruses and the genetic structure of populations [20].

The *IL10* rs1800871 CC genotype in the Alpha variant and CT genotype in the Delta variant had a relationship with COVID-19 mortality; nonetheless, there was no association between the rs1800871 polymorphism with the Omicron BA.5 variant in the current study. In contrast to the present study, a study in Mexico indicated that the *IL10* rs1800871 and rs1800872 polymorphisms among 193 COVID-19 patients were not linked to the severity of the disease. Probably, the reason for this difference is the number of examined samples, which in the current study was much more, and another reason could be the difference in ethnicity. The relationship between rs1800871 and other viral infections was shown in HBV infection. It has been demonstrated that the *IL10* rs1800871 C allele and CC genotype can increase the risk of HBV infection [29]. In addition, a higher risk of systemic lupus erythematosus in Iranian patients was observed at the *IL10* rs1800871 CC genotype [17].

The above-mentioned three SNPs’ impact on COVID-19 susceptibility might be explained by haplotype analysis. According to the obtained data of the current study, the GTA haplotype was the most common of haplotype in different SARS-CoV-2 variants. The TCG haplotype was related to COVID-19 mortality in Alpha, Delta, and Omicron BA.5 variants. In prior reports, the TCG haplotype was observed with increased IL10 production, compared to other haplotypes [30]. In this study, *IL10* haplotype distribution indicated that the frequency of the TCG haplotype among the deceased COVID-19 patients in three different SARS-CoV-2 variants was higher than in the recovered subjects.

In addition to the strength of this study in examining these polymorphisms with the death rate of COVID-19 in different variants of SARS-CoV-2, this study included some limitations. The lack of access to healthy individuals who did not have a history of COVID-19 and comparing the results with them was one of the main limitations of the study. Additionally, the results were obtained in one ethnic group, and other ethnic groups living in Iran should be examined to confirm the results. Another limitation of this study was not examining the serum level of *IL10* due to a lack of budget.

In conclusion, the *IL10* rs1800871, rs1800872, and rs1800896 polymorphism had an impact on COVID-19 infection, and these polymorphisms had different effects on various SARS-CoV-2 variants. To verify the obtained findings, further studies should be conducted on various ethnic groups.

## Data Availability

All data generated or analyzed during this study are included in this published article.
